# Influencing Factors and Cumulative Risk Analysis of Cervical Lymph Node Metastasis of Papillary Thyroid Microcarcinoma

**DOI:** 10.3389/fonc.2021.644645

**Published:** 2021-09-30

**Authors:** Yirong Yin, Xiang Xu, Liyan Shen, Wenjuan Zhao, Hongcui Diao, Chengqian Li

**Affiliations:** ^1^ Department of Endocrine and Metabolic Diseases, The Affiliated Hospital of Qingdao University, Qingdao, China; ^2^ International Medical Center, The Affiliated Hospital of Qingdao University, Qingdao, China

**Keywords:** papillary thyroid microcarcinoma, lymph node metastasis, clinicopathological features, cumulative risks, body mass index

## Abstract

**Objective:**

To explore the influencing factors and cumulative risk of lymph node metastasis (LNM) in papillary thyroid microcarcinoma (PTMC) patients.

**Methods:**

607 patients confirmed PTMC pathologically after thyroidectomy were enrolled in this retrospective study. The rate of LNM was calculated. Different clinicopathological characteristics were compared in PTMC patients with and without LNM and in different subgroups of LNM, respectively. Correlation between clinicopathological characteristics and LNM was analyzed and the cumulative risk of LNM according to different clinicopathological characteristics was calculated.

**Results:**

(1) There were 228 cases (37.56%) of PTMC combined with LNM. Compared with the non-lymph node metastasis group, the proportion of age <55 years, male, multiple foci, bilateral foci, diameter>0.5cm, extracapsular invasion, HT and intermediate-to-high risk stratification for recurrence of the LNM group was significantly increased (all *p*<0.05);(2) Multivariate logistic regression analysis showed that age <55years, male, multiple foci, diameter>0.5cm,HT were independently positively correlated with LNM (all *p <*0.05); (3) Subgroup analysis showed that women aged <55 years combined with HT and aged≥55 years combined with BMI≥25 kg/m^2^ were independently positively associated with LNM; (4) With the increase of the tumor diameter, the cumulative risk of LNM in group of age <55 years, males, and multiple foci increased gradually, and was higher than those of age≥55 years, females and single foci, respectively. (5) Among the 228 cases of LNM, the proportion of lymph nodes (LN) >5 and the positive rate of LN were both higher in male group than that in the female group. The proportion of multiple foci and HT in LLNM group was higher than that in CLNM group (all P<0.05).

**Conclusion:**

Age <55 years, males, multiple foci, diameter >0.5cm and HT were independent risk factors of LNM; HT was an independent risk factor for LNM in female <55 years old, and BMI≥25 kg/m^2^ was an independent risk factor for LNM in female ≥55 years old; The increase of tumor diameter in age <55 years, males, multiple foci, and bilateral foci increased the cumulative risk of LNM, respectively; The number of LNM and the positive rate of LNM were both higher in male, and patients with multiple foci or HT were more likely to develop into LLNM.

## Introduction

In recent years, the incidence of thyroid cancer is on the rise (1), among which papillary thyroid carcinoma (PTC) is the most frequent pathological type, accounting for about 90% (2). Previously, cervical lymph node metastasis (LNM) doesn’t affect the prognosis and overall survival of papillary thyroid microcarcinoma (PTMC) patients. However, a growing number of reports suggest that LNM may have a negative impact on the prognosis of PTMC patients recently (3, 4).It is found that (5–7) age <55 years(or <45 years), male, tumor diameter >0.5cm (or >0.6cm), multiple foci, bilateral foci, extracapsular invasion, follicular carcinoma, tumor calcification and distant metastasis are independent risk factors of LNM. Studies have also shown (8, 9) that the combination of different clinicopathological characteristics will increase the cumulative risk of poor prognosis. The relationship between Hashimoto’s Thyroiditis (HT) and LNM has been controversial. Some scholars claim that HT promotes the occurrence of LNM (10), while others believe that HT is not correlated with LNM (7).At the same time, the incidence of obesity is increasing (11).Studies have shown that high Body mass index (BMI) not only promotes the occurrence of thyroid cancer (12–14), but also leads to poor prognosis (15–17).At present, there are limited studies on the correlation between BMI and LNM in different groups of genders and ages (18). This study aims to explore the risk factors for LNM in PTMC, clarify the relationship between HT and LNM and between BMI and LNM, and calculate the cumulative risk of LNM according to different clinicopathological characteristics, so as to provide evidence for the accurate prediction of cervical lymph node metastasis.

## Materials and Methods

### Subjects

A retrospective analysis was performed on 607 patients who underwent thyroidectomy and were pathologically confirmed as PTMC in the Affiliated Hospital of Qingdao University from October 2018 to December 2019. Patients with other malignant tumors, major organ diseases, system diseases and incomplete clinical data were excluded. The basic information of 607 PTMC patients was shown in **Table 1**. There were 228 cases (37.56%) of PTMC combined with LNM. Ipsilateral lymph nodes(LN) were 222, bilateral LN were 6. The diameter of LN ranged from 0.12 to 4.50 cm, with an average of 1.02 ± 0.55cm. The number of LN ranged from 1 to 36 with a median of 7. The positive rate of LNM in resected LN was 2.00% to 100%, with a median of 33%.

**Table 1:** Basic information of 607 PTMC patients.

Index	Total
Gender	
Male	141 (23.23)
Female	466 (76.77)
Age (Year)	45.58 ± 11.75
BMI (kg/m^2^)	25.12 ± 3.29
Surgery	
Total or subtotal thyroidectomy	228 (37.56)
Thyroid gland and isthmic lobectomy	379 (62.44)
LND^※^	
CLND^※※^	607 (100)
CLND and LLND^※※※^	91 (14.99)
^131^ I	
Yes	61 (10.05)
No	546 (89.95)
HT^※※※※^	
Yes	166 (27.35)
No	441 (72.65)
Bilateral foci	
Yes	125 (20.59)
No	482 (79.41)
Tumor diameter (cm)	0.67 ± 0.23
Number of foci	1

Data were expressed as n (%), mean ± standard error (S.E.), and median. ^※^denotes lymph node dissection; ^※※^denotes central lymph node dissection; ^※※※^lateral lymph node dissection; ^※※※※^denotes the pathological results.

### Indications for Surgery

**Figure 1:**
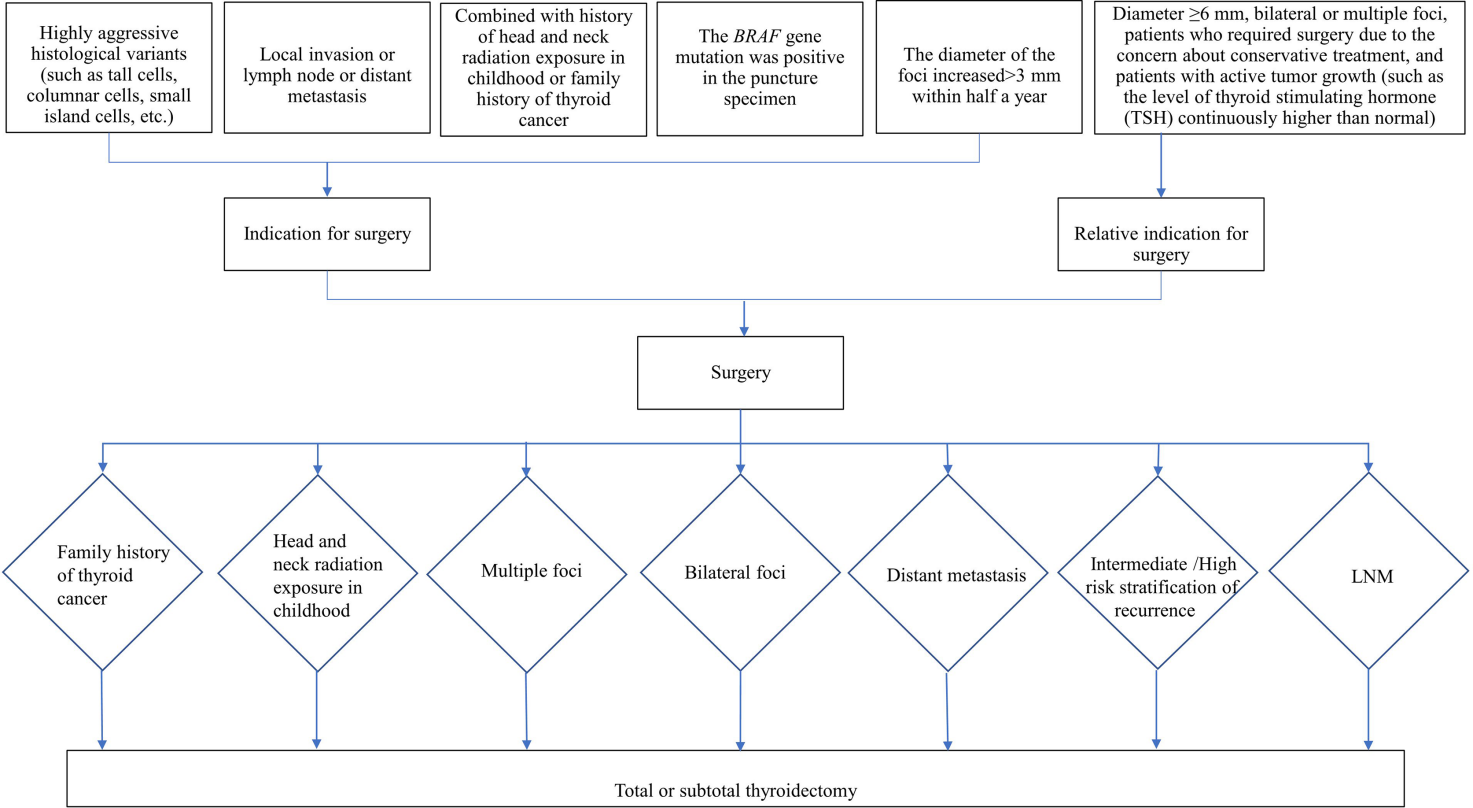
Indications for surgery and for total or subtotal thyroidectomy.

According to the *Chinese Expert Consensus on Diagnosis and Treatment of Thyroid Micropapillary Carcinoma (2016)* (19) (hereinafter referred to as the Chinese consensus) and 2015 ATA guideline (20), the indications for surgery and for surgical mode are as follows: 1. Indications for surgery are shown in **Figure 1**, 2. The choice of surgical mode is based on the imaging characteristics from two-dimensional ultrasound, risk factor assessment, and histological characteristics of the tumor (invasion, multiple foci, lymph node metastasis, etc.). The pros and cons of various surgical modes and the patient’s intention are also taken into account to make the individualized treatment plans. Indications for total or subtotal thyroidectomy are shown in **Figure 1**, and patients who doesn’t meet the criteria are underwent thyroid gland lobe and isthmus resection. 3. Indications for lymph node dissection: (1) Central lymph node dissection: ① Therapeutic central-compartment neck dissection: patients with clinically involved central nodes. ② Prophylactic central-compartment neck dissection: PTC patients with clinically uninvolved central neck lymph nodes (cN0) who have advanced primary tumors (T3b or T4) or clinically involved lateral neck nodes (cN1b); patients without LNM in preoperative ultrasound but with surgical intention under the premise of technical assurance; (2) Lateral lymph node dissection: ① Patients with LLNM confirmed by preoperative evaluation or intraoperative frozen pathological examination; ② Patients with CLNM that have extra invasion or the number of LNM is ≥3, or the foci is located in the upper pole with capsule invasion; ③ Prophylactic lateral lymph node dissection is not performed for cN0 patients.

### Measurements

Clinicopathological data of the patients were collected through the hospital database, including:① Age, gender, height, weight and surgery mode. BMI=weight (kg)/height (m)^2^. Classification standard of WHO was adopted for obesity: 18.5≤BMI<25kg/m^2^ means normal weight, 25≤BMI<30kg/m^2^ means overweight and BMI≥30kg/m^2^ means obesity; ②PTMC, HT, tumor diameter, number of foci, unilateral/bilateral foci, extracapsular invasion, distribution of LN, ipsilateral/bilateral LN, diameter of LN and number of LN were confirmed by pathology. The positive rate of LNM = the number of LNM/the total number of LNs resected. The maximum diameter of the largest foci was taken when patients have multiple foci and multiple metastases; ③ *BRAF* V600E gene detection: DNA was extracted from tumor tissue extracted by postoperative pathological wax slices, and the point mutation of *BRAF* gene V600E was detected by fluorescent polymerase chain reaction (PCR method). DNA extraction and genetic testing were performed according to DNA kit (Beijing Tiangen Biochemical Technology Co., Ltd.) and human *BRAF* proto-oncogene serine/threonine protein kinase kit (Xiamen Aide Biomedical Technology Co., Ltd.) respectively; ④ cN0 is defined as no lymph node metastasis found in preoperative ultrasound and intraoperative examination. The rate of occult lymph node metastasis = pN1 of cN0/cN0. The proportion of occult lymph nodes in the central area = pN1 of central cN0/pN1 in all central areas; ⑤ Distant metastases were confirmed by CT and/or ^131^I whole body scan (^131^I-WBS) and SPECT/CT fusion imaging.⑥ Recurrence risk was stratified according to 2015 ATA guideline (20) and TNM stage was evaluated according to the 8th edition of the system recommended by the American Joint Committee on Cancer (AJCC) (21).

### Statistical Analyses

Statistical analysis was performed using the SPSS 23.0 statistical software. The data were expressed as mean ± standard error (S.E.). Categorical data was presented as percentage and comparisons between groups were analyzed by chi-square test. Affecting factors of LNM were assessed by analyzing the differences of clinical characteristics between the lymph node metastasis and non-lymph node metastasis groups. To identify independent affecting factors of LNM, multivariate logistic regression analysis was performed by adjusting for possible confounding factors. Cumulative risk was analyzed by Kaplan-Meier curve and the difference was statistically analyzed by log-rank method. A *p*<0.05 was considered statistically significant.

## Results

### Univariate Analysis

According to the distribution and number of LNM confirmed by postoperative pathology, we calculated the rate of LNM in different distributions. There were 228 cases of PCN1, including 167 cases of CLNM, 43 cases of LLNM, 18 cases of CLNM+LLNM. The total rate of LNM, CLNM and LLNM accounted for 37.56% (228/607), 30.48% (167 + 18/607) and 10.05% (43 + 18/607), respectively. There were 514 cases of central CN0 patients diagnosed by preoperative ultrasound or intraoperative examination, including 135 cases of central PCN1 and 10 cases of central + lateral PCN1. The rate of occult CLNM was 28.21% (135 + 10/514), and the number of occult CLNM accounted for 78.38% of the total number of CLNM (135 + 10/167+18). There were 516 cases of lateral CN0, including 9 cases of lateral PCN1 and 2 cases of central + lateral PCN1, and the proportion of occult LLNM was 2.13% (9 + 2/516).

According to postoperative pathological results, 228 cases (37.56%) were divided into lymph node metastasis group and 379 cases (62.44%) into non-lymph node metastasis group. Compared with the non-lymph node metastasis group, the proportion of age <55 years, male, multiple foci, bilateral foci, diameter >0.5cm, extracapsular invasion, HT, and intermediate-to-high risk stratification for recurrence in lymph node metastasis group was significantly higher (all *p*<0.05), while the differences in BMI, *BRAF* gene mutation rate, distant metastasis, and TNM stage were not statistically significant (all *p*>0.05) (**Table 2**).

**Table 2:** Analysis of the risk factors of LNM between various clinicopathological characteristics (n %).

Index	N	LNM	*X^2^*	*P*
		[n (%)]		
Age (Year)			5.636	0.018
<55	466	187 (40.13)		
≥55	141	41 (29.08)		
Gender			10.132	0.001
Male	141	69 (48.94)		
Female	466	159 (34.12)		
BMI (kg/m^2^)			0.331	0.565
<25	321	124 (38.63)		
≥25	286	104 (36.36)		
Number of foci			37.557	0.000
Single	420	124 (29.52)		
Multiple	187	104 (55.61)		
Unilateral or bilateral foci			17.265	0.000
Unilateral	482	161 (33.40)		
Bilateral	125	67 (53.60)		
Tumor diameter (cm)			37.043	0.000
≤0.5	209	44 (21.05)		
0.6~1	398	184 (46.23)		
Extracapsular invasion			5.387	0.020
Yes	98	47 (47.96)		
No	509	181 (35.56)		
HT			4.796	0.029
Yes	166	74 (44.58)		
No	441	154 (34.92)		
*BRAF* mutation			0.363	0.547
Positive	505	187 (37.03)		
Negative	102	41 (40.20)		
Distant metastasis			2.156	0.142
Yes	7	5 (71.43)		
No	600	223 (37.17)		
Risk stratification of recurrence			236.931	0.000
Low risk	360	45 (12.50)		
Intermediate/High risk	247	183 (74.09)		
TNM stage			3.556	0.059
I-II	586	216 (36.86)		
III-IV	21	12 (57.14)		

### Independent Affecting Factors of LNM

Multivariate logistic regression analysis showed that age <55 years, male, multiple foci, diameter >0.5cm and HT were still positively correlated with LNM in PTMC patients (all P<0.05). However, bilateral foci and extracapsular invasion were not correlated with LNM in PTMC patients (all *p*>0.05) (**Table 3**).

**Table 3:** Multivariate logistic regression analyses to identify independent affecting risk factors of LNM.

Index	OR	95%CI	*p*
Age <55years	1.712	1.103~2.656	0.016
Male	1.977	1.303~3.001	0.001
Multiple foci	3.259	1.855~5.728	0.000
Bilateral foci	0.802	0.427~1.506	0.492
Diameter >0.5 cm	2.797	1.870~4.185	0.000
Extracapsular invasion	1.227	0.762~1.975	0.400
HT	1.526	1.023~2.276	0.038

### Subgroup Analyses on Females of Different Ages

353 female patients aged <55 years were divided into two groups: 129 cases (36.54%) with LNM and 224 cases (63.46%) without LNM. Univariate analysis showed that the proportion of BMI<25kg/m^2^, multiple foci, bilateral foci, diameter >0.5cm, and HT in the LNM group was significantly higher than those of the non-lymph node metastasis group (all *p*<0.05), while there were no statistically significant differences in extracapsular invasion, *BRAF* gene mutation rate, distant metastasis, risk stratification for recurrence, and TNM stage (all *p >*0.05). Multivariate analysis showed that multiple foci, diameter >0.5 cm and HT were still positively correlated with LNM (all *p*<0.05), while BMI<25kg/m^2^ and bilateral foci were not correlated with LNM (all *p*>0.05) (**Table 4**).

**Table 4:** Multivariate logistic regression analyses to identify independent affecting risk factors of PTMC combined with lymph node metastasis in female of different age groups.

	Female <55years				Female ≥55years		
	OR	95%CI	*p*		OR	95%CI	*p*
BMI<25 kg/m^2^	1.571	0.967~2.551	0.068	BMI≥25 kg/m^2^	5.542	1.762~17.434	0.003
Multiple foci	3.371	1.561~7.278	0.002	Multiple foci	3.468	0.826~14.555	0.089
Bilateral foci	0.564	0.239~1.335	0.193	Bilateral foci	1.773	0.374~8.399	0.471
Diameter >0.5 cm	2.926	1.728~4.953	0.000	Diameter >0.5 cm	3.383	1.055~10.847	0.040
HT	2.227	1.349~3.678	0.002	Extracapsular spread	2.554	0.690~9.454	0.160

Next, 113 female patients aged ≥55 years were divided into 30 cases (26.55%) with LNM and 83 cases (73.45%) without LNM. Univariate analysis showed that the proportion of BMI≥25 kg/m^2^, multiple foci, bilateral foci, diameter >0.5cm and extracapsular invasion of the LNM group was significantly higher than those of the non-lymph node metastasis group (all P<0.05), while there were no significant differences in HT, *BRAF* gene mutation rate, distant metastasis, risk stratification of recurrence, and TNM stage (all P>0.05). Multivariate analysis showed that BMI≥25 kg/m^2^ and diameter >0.5cm were still positively correlated with LNM (all *p*<0.05), while multiple foci, bilateral foci and extracapsular invasion were not correlated with LNM (all *p*>0.05) (**Table 5**).

**Table 5:** Analysis of characteristics between different subgroups of LNM (n %).

Index	N	LLNM^※^	Bilateral	Number > 5^※※^	diameter ≥ 0.2cm^※※^	positive rate ≥ 33%^※※※^
Age (Year)						
<55	187	47(25.13)	3(1.60)	41(21.93)	171(91.44)	108(57.75)
≥55	41	14(34.15)	3(7.32)	7(17.07)	38(92.68)	24(58.54)
Gender						
Male	69	20(28.99)	2(2.90)	21 (30.43)*	65(94.20)	49 (71.01)*
Female	159	41(25.79)	4(2.52)	27(16.98)	144(90.57)	83(52.20)
Number of foci						
Single	124	26(20.97)	2(1.61)	23(18.55)	112(90.32)	75(60.48)
Multiple	104	35 (33.65)*	4(3.85)	25(24.04)	97(93.27)	57(54.81)
Tumor diameter (cm)						
≤0.5	44	8(18.18)	0(0.00)	5(11.36)	40(90.91)	23(52.27)
0.6~1	184	53(28.80)	6(3.26)	43(23.37)	169(91.85)	109(59.24)
HT						
Yes	74	26 (35.14)*	4(5.41)	16(21.62)	70(94.59)	37(50.00)
No	154	35(22.73)	2(1.30)	32(20.78)	139(90.26)	95(61.69)
Total	228	61	6	48	209	132
X^2^ = 4.645^a^, 3.927^b^, 5.240^c^, 6.987^d^						
p=0.031^a^, 0.048^b^, 0.022^c^, 0.008^d^						

^※^denotes LLNM and/or CLNM; ^※※^The 2015 ATA guideline proposed that the diameter of LN<0.2 cm and the number of LN ≤ 5 were indicators for low-risk recurrence risk stratification, so patients were grouped by them accordingly. ^※※※^Patients were grouped by median; ^a,b,c,d^ indicate the comparison of differences in the distribution of LNM between groups of single and multiple foci, the comparison of differences in the distribution of LNM between groups with or without HT, the comparison of differences in the number of LNM between groups of different genders and the comparison of differences in the positive rate of LNM between groups of different genders. *indicates comparison between two groups with p < 0.05.

### Subgroup Analyses on Males of Different Ages

113 male patients aged <55 years were divided into 58 cases (51.33%) with LNM and 55 cases (48.67%) without LNM. Univariate analysis showed that the proportion of multiple foci, bilateral foci, diameter >0.5 cm, and intermediate-to-high risk stratification for recurrence of the LNM group was significantly higher than those of the non-lymph node metastasis group (all *p*<0.05), and there were no statistically significant differences in BMI≥25 kg/m^2^, extracapsular invasion, HT, *BRAF* gene mutation rate, distant metastasis, and TNM stage (all *p*<0.05). Multivariate analysis showed that multiple foci and diameter >0.5cm were still positively correlated with LNM (all *p*<0.05), while the bilateral foci were not correlated with LNM (P>0.05).

28 male patients aged ≥55 years were divided into 11 cases (39.29%) with LNM and 17 cases (60.71%) without LNM. Univariate analysis showed that the proportion of bilateral distribution, intermediate-to-high risk stratification for recurrence, and III/IV stage of TNM were significantly higher than those of non-lymph node metastasis group (all *p*<0.05), while there were no statistical significances in BMI≥25 kg/m^2^, single foci, diameter>0.5cm, extracapsular invasion, HT, *BRAF* gene mutation rate, and distant metastasis (all *p*>0.05). Multivariate analysis showed that the bilateral foci was not significantly related to LNM (*p*>0.05).

### Cumulative Risk of LNM

**Figure 2:**
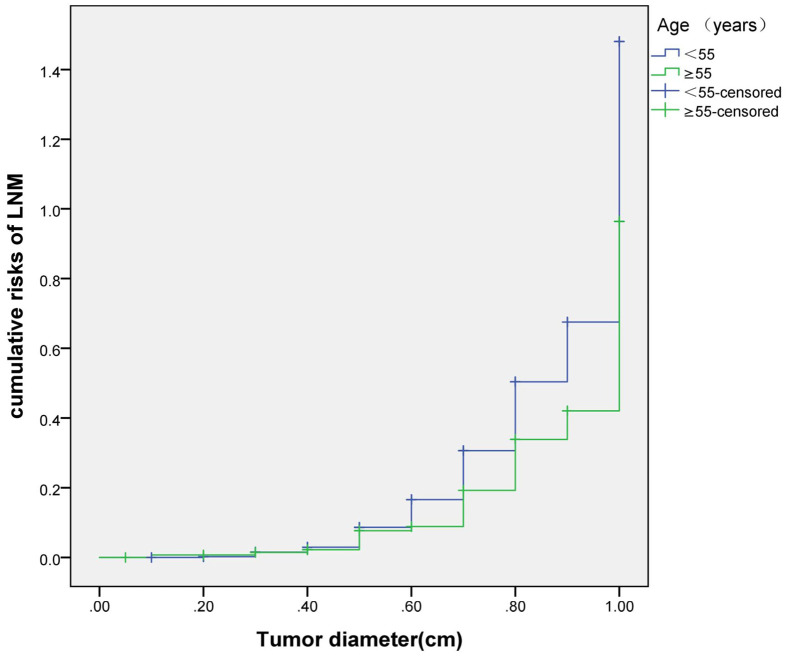
Cumulative risks of LNM according to age and tumor diameter.

The cumulative risk of LNM was calculated by combining age, gender, single/multiple foci with tumor diameter, respectively. The results showed that with the increase of tumor diameter, the risk of LNM in each combination also had a trend of elevation. Besides, the risk of LNM in group of age <55 years, males and multiple foci was significantly higher than those of age ≥55 years, females and single foci, respectively (*p* were0.024, 0.013, 0.001, 0.038). When compared at the same tumor diameter, the risk of LNM in the former group was significantly higher than those of the latter group. However, the cumulative risk of LNM was not statistically significant when calculated by tumor diameter in combination with either BMI, extracapsular invasion, HT or *BRAF* gene mutation (**Figures 2** **–** **4**).

**Figure 3:**
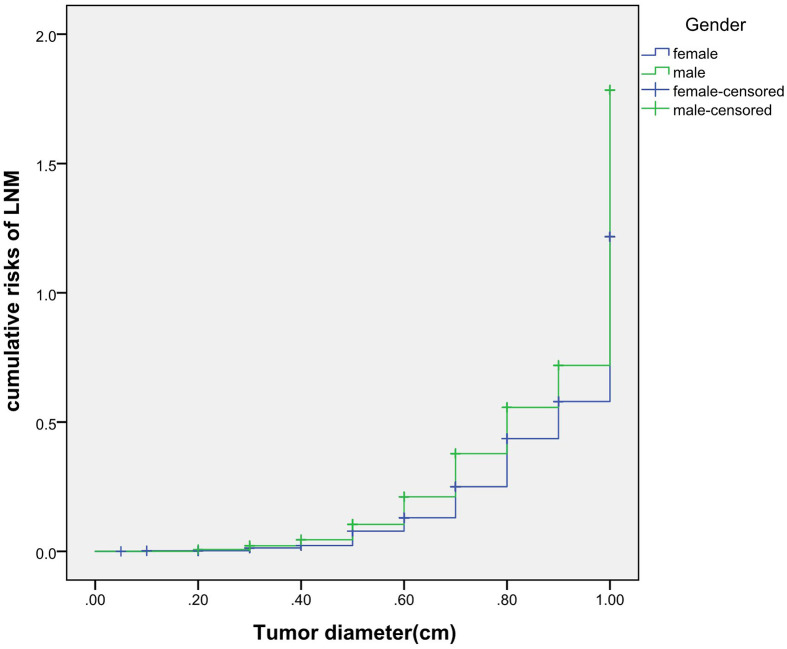
Cumulative risks of LNM according to gender and tumor diameter.

**Figure 4:**
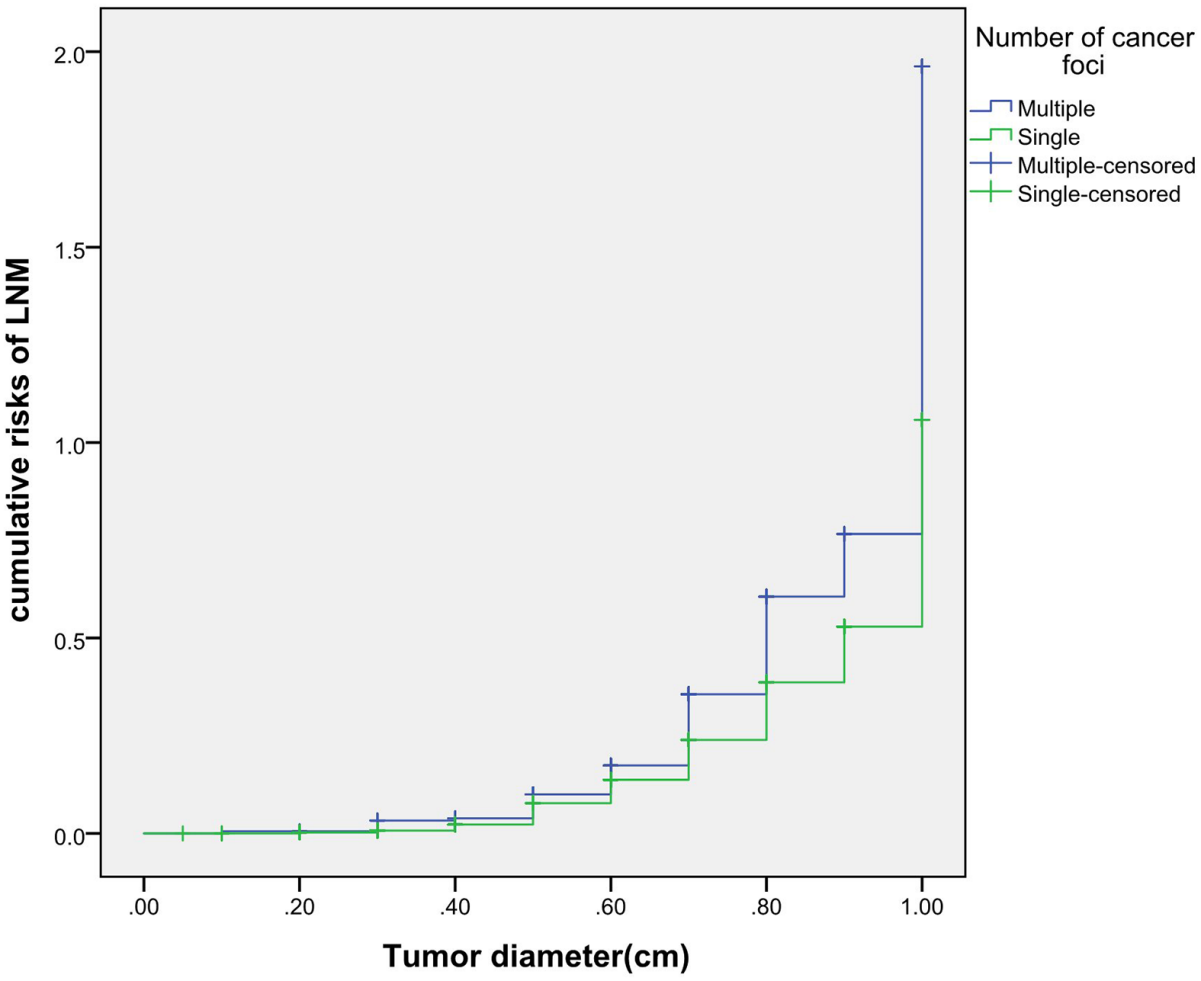
Cumulative risks of LNM according to number of cancer foci and tumor diameter.

### Subgroup Analyses on LNM of Different Characteristics

228 patients with LNM were grouped according to age, gender, diameter of foci, number of foci and whether combined with HT. The differences in different distribution of LNM, ipsilateral/bilateralism and positive rate of LNM were compared. The results showed that the proportion of number of LN >5 and positive rate of LN ≥33% in male was higher than that of female, respectively; the proportion of LLNM in multiple foci and HT group was higher than that of single foci and non-HT group, respectively; there was no significant difference in other indicators of each group (all *p*>0.05) (**Table 4**). Further multivariate logistic regression analysis showed that multiple foci was an independent risk factor for LLNM (*p* =0.044, 95%CI: 1.015-3.360).

## Discussion

The incidence of PTMC is on the rise, accounting for more than 50% of new cases of thyroid cancer (22). It is generally believed that PTMC is small in diameter and inert with a good prognosis. Therefore, observational treatment with active monitoring can be considered for extremely low risk PTMC. However, plenty scholars believe that tumor size can only represent biological morphology, and cannot completely represent the low-risk of progression. The rate of CLNM of ​​PTMC is high, and distant metastasis may even occur. Some studies have shown that the total lymph node metastasis rate of PTMC is 28%~64% (23–25), and the rate of CLNM can reach 38.5%. Ito et al. (26) find that the rate of LNM confirmed by pathological examination amounts to 40% when preventive CND (central neck dissection, CND) is performed on PTMC. Such a high rate of LNM cannot be ignored. Depending on the survey (27), the recurrence of central lymph nodes accounted for 80.0% of the total recurrences in PTMC. Wada et al. (23) find that the incidence of CLNM in the therapeutic CND and preventive CND groups is 95.8% and 60.9% in PTMC, while the recurrence rates of lymph nodes in the two groups are 16.7% and 0.4%. Both groups had a higher incidence of CLNM, while preventive CND reduced the recurrence of lymph node, which means that once the LNM is controlled, the local recurrence is controlled. However, some scholars believe that CLNM does not affect the prognosis due to the slow development of PTMC and lymph node dissection may increase the risk of recurrent laryngeal nerve injury and permanent hypoparathyroidism (28). Therefore, whether or not to perform the preventive central lymph node dissection is still the focus of controversy among scholars. We conduct a retrospective analysis of 607 PTMC, finding that the total rate of LNM is 37.56%, of which CLNM is 30.84%, and LLNM is 10.05%; the rate of occult CLNM is 28.21%, the number of occult CLNM accounted for 78.38% of the total number of CLNM, and the rate of occult LLNM is only 2.13%. This suggests that the incidence of LNM in PTMC is high and the central area is the most common area, especially in the form of occult metastasis. We also find that the proportion of middle/high risk of recurrence stratification in LNM group was higher than that in the non-metastatic group, suggesting that LNM increases the risk of recurrence. This result supports the recommendation made by the Chinese Consensus (19), which supports that preventive lymph node dissection in area VI helps reduce recurrence and avoid reoperation under the premise of technical guarantee and the patient’s consent. Moreover, the rate of LLNM and occult LLNM is low, suggesting that it is not necessary for patients with lateral cN0 to perform prophylactic lateral lymph node dissection which is consistent with the recommendations of the 2015 ATA guideline.

Currently, age<55 years (or <45 years), male, diameter>0.5cm (or>0.6cm), multiple foci, bilateral foci, extracapsular invasion, follicular carcinoma, tumor calcification and distant metastasis are independent risk factors of LNM (5–7). We conducted a retrospective study of 607 patients with PTMC, and found that the incidence of LNM was37.56%, within the range of published data. Additionally, the proportion of age <55 years, male, multiple foci, bilateral foci, diameter >0.5cm, HT and intermediate-to-high risk stratification for recurrence of LNM group was significantly higher than those of the non-lymph node metastasis group. Multivariate logistic regression analysis found that age <55 years, male, multiple foci, diameter >0.5cm, and HT were positively correlated with lymph node metastasis, and the OR values ​​were1.712, 1.997, 3.259, 2.797 and 1.526 respectively, which were consistent with previous studies. It shows that the above factors are independent risks of LNM in PTMC patients, and LNM increases the high risk of recurrence. In addition, this study does not find that extracapsular invasion, *BRAF* gene mutation rate, distant metastasis and TNM stage are associated with LNM, which is similar to some previous studies (29, 30), suggesting that these are not the main risk factors of LNM, and LNM does not affect TNM stage. However, many studies about long-term survival and mortality are based on active treatment currently, and the prospective data of routine prophylactic CLND is absence. Whether it is necessary for patients with PTMC to perform routinely prophylactic CLND requires more and longer research data to illustrate. The results of recurrence and prognosis in this study are still in the process of follow-up due to the relatively good prognosis and a high 5-year survival rate.

In recent years, the incidence of thyroid cancer and obesity has increased (1, 11). Epidemiological investigations have found that high BMI is involved in a variety of tumors such as gastric cancer, colorectal cancer, and breast cancer in postmenopausal women (31, 32). A meta-analysis shows that overweight and obesity can increase the likelihood of thyroid cancer by 25% and 55%, respectively. For every 5kg/m^2^ increase in BMI, the risk of thyroid cancer increases by 30% (33). Researchers at worldwide in recent years have also showed similar results (12–14).

The relationship of BMI and thyroid cancer progression is controversial. Some studies believe that low BMI (<22kg/m^2^) promotes the occurrence of LNM (34), while some believe that higher BMI is not related to the aggressive characteristics (35, 36). Most studies agree that high BMI promotes poor prognosis of thyroid cancer, such as promoting extrathyroidal invasion, advanced TNM stage, multiple foci, LNM and tumor growth (15–17).Kim’s (18)study showed that higher BMI is an independent predictor of extrathyroidal invasion and multiple foci in female PTC patients, but not a predictor of males, suggesting that BMI is related to poor prognosis but there are gender differences. This study does not find that BMI≥25kg/m^2^ is related to LNM in PTMC patients, but subgroup analysis finds that BMI≥25kg/m^2^ is an independent risk factor of LNM in female ≥55 years, with an OR value of 3.790, and this correlation is meaningless in female<55 years, male<55 years and male ≥55 years (all *p*>0.05). Therefore, for female ≥55 years and BMI ≥25 kg/m^2^, weight loss should be emphasized to reduce the risk of LNM in PTMC patients. In addition, there are only 28 cases in the male ≥55 years in this study, and multivariate analysis does not find risk factors of LNM, which may be related to the small number of cases and the deviation of the results. The sample size should be expanded in the future for further research.

The mechanisms that high BMI promotes the occurrence and development of thyroid cancer mainly include hyperinsulinemia-related pathway and non-hyperinsulinemia-related pathway (37, 38). Long-term obesity leads to insulin resistance and hyperinsulinemia. Insulin, as a cell growth factor, activates phosphatidylinositol 3-kinase/protein kinase B (PI3K/AKT) and mitogen-activated protein kinase (MAPK) signaling pathways, prompts angiogenesis and prompts tumor proliferation (39); non-hyperinsulinemia pathways include the estrogen or androgen imbalance caused by increased aromatase activity in adipose tissue of obese patients, the enhanced effects of adipokines and inflammatory factors, and increased thyroid-stimulating hormone (TSH) levels, etc. (40–43). In addition, the effect of BMI on thyroid cancer is influenced by gender and age. According to report (44), the average age of menopause in Chinese female is about 49-51 years. The change of endocrine and metabolic balance caused by menopause can easily cause obesity (45). Besides, the secretion of androstenedione and the activity of aromatase in postmenopausal obese patients are enhanced, which converts androstenedione secreted by the ovaries and adrenal glands into estrogen, leading to the proliferation of cancer cells and angiogenesis (40). This may be one of the reasons for the occurrence of LNM in female aged ≥55 years and BMI≥25kg/m^2^ in this study.

The relationship between HT and LNM has always been controversial. Zhang believes that HT promotes the occurrence of LNM (10), while Xue believes that HT has nothing to do with LNM (7). At present, the exact mechanism of how HT participates in the progress of PTMC is not very clear. Most studies believe that HT promotes the occurrence of LNM through its high expression of TPOAb, TGAb and TSH (46, 47). This study finds that the HT promotes the occurrence of LNM in PTMC. And further subgroup analysis of gender and age shows that HT is independent risk factor for LNM in female patients <55 years only, suggesting that female aged <55 years with high serum levels of TPOAb and TGAb should be more alert to LNM, and prophylactic cervical lymph node dissection should be performed when necessary.

Previously, many studies focus on the independent risk factors of LNM in PTMC patients (5–7), but there are no relevant reports on assessing cumulative risk of LNM according to tumor diameter and different clinicopathological characteristics. At present, it has been reported that the combination of different clinicopathological features increases the cumulative risk of distant metastasis. Gao (8)finds that the lymph node metastasis rate>26.43% is an independent predictor of PTMC distant metastasis. With the increase of the lymph node metastasis rate, the cumulative risk of distant metastasis in the number of LNM≥15 group is significantly higher than the number of LNM< 15 group. Min (9)uses tumor diameter, number of metastatic lymph nodes, and metastasis grade to predict the risk of distant metastasis in 1,700 patients with PTC. The results show that with the increase of tumor diameter, the cumulative risk of distant metastasis increases most in the number of lymph nodes> 20 group, followed by in the number of lymph nodes 6-20 group; similarly, the pN1b group is the most elevated group followed by the pN1a group. These results suggest that the combination of different pathological characteristics can promote the accumulation of risk and aggravate the poor prognosis. This study also finds that with the tumor diameter increases, the cumulative risk of LNM in group of age < 55 years, males and multiple foci increases gradually, and is higher than those ofage≥55 years, females and single foci, respectively. This result suggests that the combination of the above-mentioned risk indicators contributes to LNM in PTMC patients. Therefore, for a certain PTMC patient, a comprehensive analysis of its clinicopathological characteristics can predict the risk of LNM more comprehensively, effectively avoiding the limitation of predicting LNM by a single index.

We further analyze the influence of independent risk factors for LNM on the characteristics of LNM. The results show that the number of LNM and positive rate of LNM were increased in male group, the multiple foci and HT were increased in LLNM group and the multiple foci was an independent risk factor for LLNM, suggesting that preoperative screening should be strengthened in clinical work for patients with multiple foci and HT in order to be alert to the possibility of LLNM, and the thoroughness of intraoperative lymph node dissection should be considered for male patients to improve the cure rate also.

Recent studies have shown that factors such as the number, diameter and extranodal invasion of LNM also significantly affect the recurrence of the disease. Wu et al. (48) and Park et al. (49) confirmed that the number of LNM is one of the independent predictors of prognosis. Recurrence-free survival rate was lower in patients with more than 3 metastatic lymph nodes than in patients with less than 3 metastatic lymph nodes (89.2% *vs* 98.5%). The rate of local recurrence in DTC is on the rise due to the increase of LNM number and LNM diameter (50, 51). In addition, extra-lymph node involvement is among the independent predictors of recurrence in DTC (HR=12.597), and its 10-year recurrence rate can reach as high as 38% (48, 52). Based on the above evidence, 2015 ATA guideline has included the number of LNM, involved diameter of LNM, and extranodal invasion as weighting factors into the recurrence risk stratification system. This updated content provides rationality of making a more accurate and individualized plan for postoperative treatment and follow-up. Based on the evaluation system of the new guideline (20), the number and diameter of metastatic lymph nodes were taken into account when stratifying the risk of recurrence in our study. Risk stratification management is of great value to reduce recurrence and improve prognosis. However, long-term follow-up is still needed not only to evaluate the characteristics of LNM, but also further explore the relationship between the characteristics of different LNM and the recurrence as well as the prognosis of PTMC.

In summary, age <55 years, males, multiple foci, diameter >0.5cmand HT are independent risk factors of LNM in PTMC; HT is the independent risk factor of LNM for female aged <55 years, just like BMI≥25kg/m^2^ for female aged ≥55 years; LNM increases the risk of recurrence, but does not affect the prognosis; the increase of tumor diameter in age <55 years, males and multiple foci increased the cumulative risk of LNM, respectively; gender, multiple foci and HT affect the characteristics of LNM. Therefore, the combination of different clinicopathological characteristics can more comprehensively predict the risk of LNM. It can be seen that the risk factors of LNM should be fully assessed for patients with PTMC before surgery, and LNM should be vigilant; for patients with LNM, attention should be paid to the risk assessment of postoperative pathological metastasis lymph node characteristics, and rational treatment plans can be formulated for patients, which can not only effectively reduce recurrence and the risk of death but also can avoid over-treatment.

## Data Availability Statement

The raw data supporting the conclusions of this article will be made available by the authors, without undue reservation.

## Author Contributions

All authors were involved conception and design of this manuscript. YY performed the data analysis and wrote the manuscript. LS and HD gave helpful suggestions. XX, WZ, and CL revised the manuscript. All authors contributed to the article and approved the submitted version.

## Funding

The present study was supported by Natural Science Foundation of Shandong Province, China (Project No. ZR2016HM29).

## Conflict of Interest

The authors declare that the research was conducted in the absence of any commercial or financial relationships that could be construed as a potential conflict of interest.

## Publisher’s Note

All claims expressed in this article are solely those of the authors and do not necessarily represent those of their affiliated organizations, or those of the publisher, the editors and the reviewers. Any product that may be evaluated in this article, or claim that may be made by its manufacturer, is not guaranteed or endorsed by the publisher.
